# Genetic characterization of *Cryptosporidium* spp. and *Giardia duodenalis* in dogs and cats in Guangdong, China

**DOI:** 10.1186/s13071-019-3822-z

**Published:** 2019-11-29

**Authors:** Jiayu Li, Xiaoyu Dan, Kexin Zhu, Na Li, Yaqiong Guo, Zezhong Zheng, Yaoyu Feng, Lihua Xiao

**Affiliations:** Key Laboratory of Zoonosis of Ministry of Agriculture, College of Veterinary Medicine, South China Agricultural University, Guangzhou, 510642 China

**Keywords:** *Cryptosporidium* spp., *Giardia duodenalis*, Genotype, Risk factors

## Abstract

**Background:**

There are only limited number of reports on molecular epidemiology of *Cryptosporidium* spp. and *Giardia duodenalis* in dogs and cats in China. This study was conducted to assess the infection rates, genetic identity, and public health potential of these parasites in dogs and cats in Guangdong, China.

**Methods:**

PCR and sequence analyses were used to identify and genotype *Cryptosporidium* spp. and *G. duodenalis* in fecal samples from 641 dogs and 418 cats in Guangdong. Chi-square test and odds ratio analysis were used to compare the occurrence rates of these pathogens and identify risk factors for infection.

**Results:**

The overall infection rates of *Cryptosporidium* spp. and *G. duodenalis* were 6.9% (44/641) and 9.4% (60/641) in dogs, and 6.2% (26/418) and 3.6% (15/418) in cats. Purebred cats (12.4%; *χ*^2^ = 5.110, OR = 2.8, *P* = 0.024) and dogs (10.8%; *χ*^2^ = 5.597, OR = 4.8, *P* = 0.018) were more likely to be infected by *Cryptosporidium* spp. and *G. duodenalis*, respectively. Dogs (12.0%; *χ*^2^ = 7.589, OR = 2.6, *P* = 0.006) and cats (13.6%; *χ*^2^ = 8.235, OR = 3.5, *P* = 0.004) under 6 months had significantly higher infection rates of *Cryptosporidium* spp. than older animals. Household (13.9%; *χ*^2^ = 10.279, OR = 2.6, *P* = 0.008) and pet shop dogs (11.0%; *χ*^2^ = 7.182, OR = 2.0, *P* = 0.048) had higher occurrence of *Cryptosporidium* spp., as was the case for *G. duodenalis* occurrence in experimental dogs (13.4%; *χ*^2^ = 9.223, OR = 1.9, *P* = 0.017). *Cryptosporidium canis* (*n* = 42), *C. muris* (*n* = 1) and *Cryptosporidium* rat genotype IV (*n* = 1) were identified in dogs, while *C. felis* (*n* = 21), *C. parvum* (*n* = 3), *C. muris* (*n* = 1) and *Cryptosporidium* rat genotype IV (*n* = 1) were identified in cats. In contrast, the canine-specific assemblages C (*n* = 27) and D (*n* = 26) and the feline-specific assemblage F (*n* = 14) were almost exclusively the only genotypes of *G. duodenalis* in dogs and cats, respectively. There was no significant difference in infection rates of *Cryptosporidium* spp. and *G*. *duodenalis* between diarrheal and non-diarrheal pets.

**Conclusions:**

While domestic pets in Guangdong are infected with zoonotic *Cryptosporidium* species, they are mainly infected with host-specific *G. duodenalis* genotypes. Risk factors for infections differ between *Cryptosporidium* spp. and *G. duodenalis* and between dogs and cats.
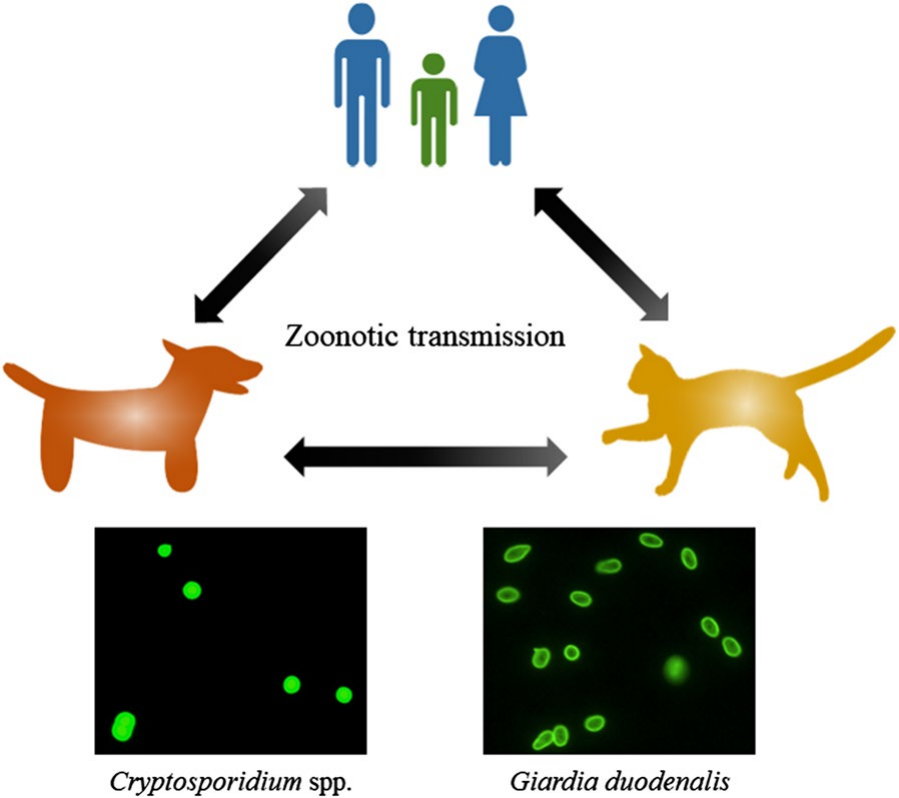

## Background

*Cryptosporidium* spp. and *Giardia duodenalis* are important protozoan parasites that inhabit the gastrointestinal tract of humans and other vertebrates. Diarrhea is the main clinical symptom of cryptosporidiosis and giardiasis. Humans acquire these two pathogens through contact with infected persons and animals, or consuming contaminated food or water [1, 2]. Among the ~40 known *Cryptosporidium* species, *C. hominis*, *C. parvum*, *C. meleagridis*, *C. canis* and *C. felis* are the most common species in humans [3]. Similarly, among the eight common genotypes (A to H) of *G. duodenalis*, only assemblages A and B are major human pathogens [2].

*Cryptosporidium* spp. and *G. duodenalis* are commonly detected in dogs and cats worldwide [4, 5]*. Cryptosporidium canis* and *C. felis* are major *Cryptosporidium* species in dogs and cats respectively, but *C. hominis*, *C. parvum*, *C. muris* and *C. ubiquitum* have been occasionally detected in these animals [6–10]. Similarly, dog-adapted assemblages C and D, and cat-adapted assemblage F are the dominant *G. duodenalis* genotypes in these animals, although zoonotic assemblages A and B have been identified in some studies [2, 11].

Limited data are available on the transmission of *Cryptosporidium* spp. and *G. duodenalis* in dogs and cats in China. The reported infection rates of *Cryptosporidium* spp. range from 1.6% to 10.5%, with *C. canis* and *C. felis* being identified as the dominant *Cryptosporidium* species in dogs and cats, respectively. In contrast, the infection rates of *G. duodenalis* were reported to range from 1.9 to 26.2%, with assemblages A, B, C, D and E being identified in dogs and assemblage F in cats [9, 11–21]. The risk factors involved in the acquisition of cryptosporidiosis and giardiasis have rarely been examined in these studies.

Guangdong Province has the largest populations of humans (111.69 million in 2017) [22] and pets (10.62% of the > 100 million pets in the country in 2015 were in Guangdong [23] in China. The subtropical climate and abundant rainfall provide a favorable environment for the transmission of waterborne pathogens such as *Cryptosporidium* spp. and *G. duodenalis*. Both cryptosporidiosis and giardiasis are known to be common in AIDS patients and diarrheic children in Guangdong, China [24, 25]. Several studies have also reported the prevalence of *G. duodenalis* in dogs and cats in the province [12, 15, 18].

Thus far, there are no systematic studies of *Cryptosporidium* spp. in dogs and cats in the province. As children in China are sometimes infected with several zoonotic *Cryptosporidium* species (*C. canis* and *C. felis*) that are traditionally associated with pets [26, 27], we examined in this study the occurrence and identity of *Cryptosporidium* spp. and *G. duodenalis* in dogs and cats in Guangdong for the assessment of the zoonotic potential of these pathogens.

## Methods

### Sample collection

**Fig. 1:**
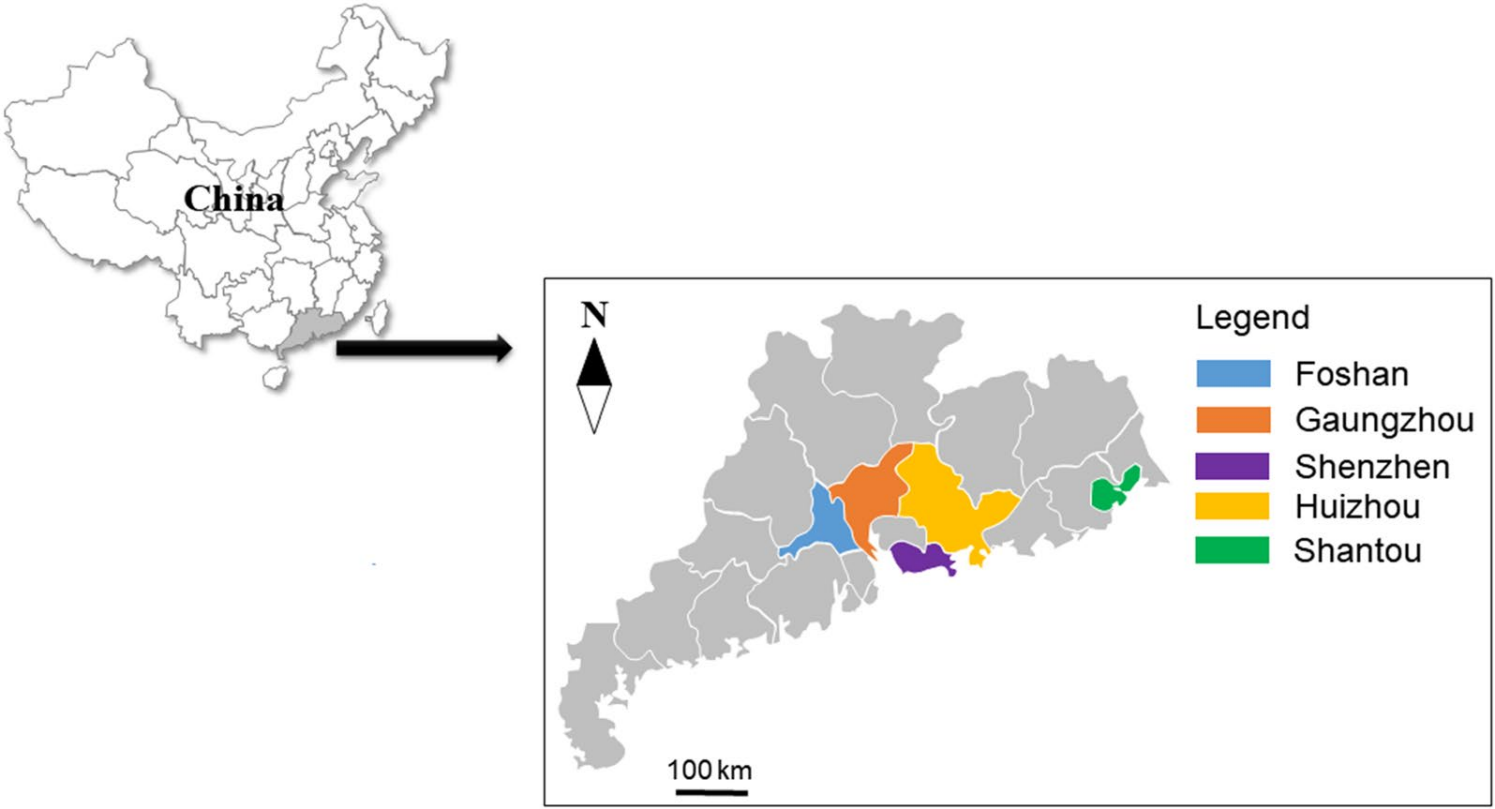
Cities in Guangdong, southern China examined for *Cryptosporidium* spp. and *Giardia duodenalis* in dogs and cats

From July 2017 to August 2018, 1059 fecal samples were collected from dogs and cats in five cities of Guangdong (Fig. 1). Among them, 641 were from dogs of various living settings, including households (*n* = 79), veterinary clinics (*n* = 109), pet shelters (*n* = 134), pet shops (*n* = 118) and a research center (*n* = 201). Simultaneously, 418 fecal samples were collected from cats in households (*n* = 49), veterinary clinics (*n* = 130), pet shelters (*n* = 132), pet shops (*n* = 27), and strays (*n* = 80) in these cities. The animals were divided into two age groups: ≤ 6 months (125 dogs and 66 cats); and > 6 months (402 dogs and 299 cats), with 114 dogs and 53 cats of unknown age. In addition, we recorded information on the sex (291 and 129 female dogs and cats, 191 and 163 male dogs and cats, respectively, and 159 dogs and 126 cats of unknown sex), breed (446 and 89 purebred dogs and cats, 82 and 187 mixed-breed dogs and cats, respectively, and 113 dogs and 142 cats of unknown breeds) and clinical signs (17 and 19 diarrheic dogs and cats, and 624 and 399 non-diarrheic dogs and cats, respectively) of the animals as conditions permitted. Each fecal sample was placed into a 50 ml plastic centrifuge tube with 2.5% potassium dichromate, transferred to the laboratory, and stored at 4 °C for less than two weeks before DNA extraction.

### DNA extraction and PCR analysis

Each fecal sample was washed twice with distilled water by centrifugation. DNA was extracted from the washed fecal materials using a Fast DNA SPIN Kit for Soil (MP Biomedicals, Santa Ana, CA, USA). The extracted genomic DNA was stored at -20 °C until use. A nested PCR targeting the small subunit (*SSU*) rRNA gene was employed to detect *Cryptosporidium* spp. [28], while PCR assays targeting the β-giardin (*bg*) [29], glutamate dehydrogenase (*gdh*) [30] and triosephosphate isomerase (*tpi*) [31] genes were employed to detect *G. duodenalis.* An ~850-bp fragment of 60 kDa glycoprotein (*gp60*) gene was amplified to identify the subtype of *C. parvum* [32]. Each sample was analyzed at least twice by PCR at each genetic locus, with both negative and positive controls being included in each PCR analysis. The secondary PCR products were analyzed by 1.5% agarose electrophoresis.

### Sequence analysis

All secondary PCR products of the expected size were sequenced on an ABI3730 autosequencer by the Sangon Biotech (Shanghai, China) in both directions using the PCR primers. The DNA sequences obtained were assembled using ChromasPro 1.5 (http://www.Technelysium.com.au/ChromasPro.html) and edited using BioEdit 7.1.3.0 (http://www.mbio.ncsu.edu/BioEdit/bioedit.html). They were aligned with reference sequences of each locus downloaded from GenBank using Clustal X 2.1 (http://www.clustal.org/) to determine the identity of *Cryptosporidium* species and *G. duodenalis* genotypes.

### Statistical analysis

Differences in infection rates of *Cryptosporidium* spp. and *G. duodenalis* in dogs and cats were compared between sexes, breeds, age groups, living conditions and clinical signs using the Chi-square test implemented in SPSS 20.0 version (IBM Inc., Chicago, IL, USA). Odds ratios (OR) and their 95% confidence intervals (95% CI) were calculated to identify risk factors involved in the acquisition of these pathogens. Differences were considered significant at *P* < 0.05.

## Results

### Occurrence and risk factors of *Cryptosporidium* infection in dogs and cats

*Cryptosporidium* spp. were detected by PCR in 44 (6.9%) of the 641 canine samples and 26 (6.2%) of the 418 feline samples (Tables 1 and 2). Odds ratios analysis identified some risk factors involved in the transmission of *Cryptosporidium* spp. in dogs and cats. Dogs (12.0%; *χ*^2^ = 7.589, OR = 2.6, *P* = 0.006) and cats (13.6%; *χ*^2^ = 8.235; OR = 3.5; *P* = 0.004) aged under 6 months were at higher risk of *Cryptosporidium* infection. Purebred cats were more susceptible to *Cryptosporidium* (12.4%; OR = 2.8; *P* = 0.024) infection. Household (13.9%; *χ*^2^ = 10.279; OR = 2.6; *P* = 0.008) and pet shop dogs (11.0%; *χ*^2^ = 7.182; OR = 2.0; *P* = 0.048) were more likely to be infected by *Cryptosporidium* spp. In contrast, there were no significant impacts on infection rates of *Cryptosporidium* spp. by sex or breed of dogs, and sex and living condition of cats (Tables 1 and 2).

**Table 1:** Infection rates of *Cryptosporidium* spp. and *Giardia duodenalis* in dogs by sex, breed, age, sample source and clinical signs

Variable	*n*	*Cryptosporidium* spp.			*G. duodenalis*		
		No. positive (%)	OR (95% CI)	*P*-value	No. positive (%)	OR (95% CI)	*P*-value
Sex							
Female	291	22 (7.6)	1.7 (0.7–3.7)	0.213	36 (12.4)	2.1 (1.1–4.2)	0.029*
Male	191	9 (4.7)	0.6 (0.3–1.3)		12 (6.3)	0.5 (0.2–0.9)	
Unknown	159	13 (8.2)			12 (7.5)		
Breed							
Purebred	446	29 (6.5)	1.1 (0.4–2.9)	0.891	48 (10.8)	4.8 (1.1–20.3)	0.018*
Mixed-breed	82	5 (6.1)	0.9 (0.3–2.5)		2 (2.4)	0.2 (0–0.9)	
Unknown	113	10 (8.8)			10 (8.8)		
Age (months)							
≤ 6	125	15 (12.0)	2.6 (1.3–5.3)	0.006**	14 (11.2)	1.3 (0.7–2.5)	0.455
> 6	402	20 (5.0)	0.4 (0.2–0.8)		36 (9.0)	0.8 (0.4–1.5)	
Unknown	114	9 (7.9)			10 (8.8)		
Sample source							
Household	79	11 (13.9)	2.6 (1.3–5.4)	0.008**	8 (10.1)	1.1 (0.5–2.4)	0.803
Pet shop	118	13 (11.0)	2.0 (1.0–3.9)	0.048*	12 (10.2)	1.1 (0.6–2.2)	0.738
Pet shelter	134	9 (6.7)	1.0 (0.5–2.1)	0.939	10 (7.5)	0.7 (0.4–1.5)	0.397
Research center	201	7 (3.5)	0.4 (0.2–0.9)	0.022*	27 (13.4)	1.9 (1.1–3.3)	0.017*
Veterinary clinic	109	4 (3.7)	0.5 (0.2–1.3)	0.148	3 (2.8)	0.2 (0.1–0.8)	0.009**
Clinical signs							
Diarrheic	17	2 (11.8)	1.8 (0.4–8.3)	0.418	2 (11.8)	1.3 (0.3–5.8)	0.730
Non-diarrheic	624	42 (6.7)	0.5 (0.1–2.4)		58 (9.3)	0.8 (0.2–3.4)	
Total	641	44 (6.9)			60 (9.4)		

*Abbreviation*: n; total number of samples

* *P* < 0.05, ** *P* < 0.01

**Table 2:** Infection rates of *Cryptosporidium* spp. and *Giardia duodenalis* in cats by sex, breed, age, sample source and clinical signs

Variable	*n*	*Cryptosporidium* spp.			*G. duodenalis*		
		No. positive (%)	OR (95% CI)	*P*-value	No. positive (%)	OR (95% CI)	*P*-value
Sex							
Female	129	11 (8.5)	1.4 (0.6–3.5)	0.433	0	112.2 (0.2–5.7 × 10^4^)	0.001**
Male	163	10 (6.1)	0.7 (0.3–1.7)		9 (5.5)	0 (1.7×10^−5^–4.6)	
Unknown	126	5 (4.0)			6 (4.8)		
Breed							
Purebred	89	11 (12.4)	2.8 (1.1–7.0)	0.024*	1 (1.1)	1.1 (0.1–11.7)	0.968
Mixed-breed	187	9 (4.8)	0.4 (0.1–0.9)		2 (1.1)	1.0 (0.1–10.6)	
Unknown	142	6 (4.2)			12 (8.4)		
Age (months)							
≤ 6	66	9 (13.6)	3.5 (1.4–8.5)	0.004**	2 (3.0)	1.0 (0.2–4.8)	0.993
> 6	299	13 (4.3)	0.3 (0.1–0.7)		9 (3.0)	1.0 (0.2–4.7)	
Unknown	53	4 (7.5)			4 (7.5)		
Sample source							
Household	49	1 (2.0)	0.3 (0.0–2.2)	0.198	1 (2.0)	0.6 (0.1–4.3)	0.573
Pet shop	27	3 (1.1)	2.0 (0.6–7.1)	0.277	0	0.1 (2.0 × 10^−4^–50.6)	0.370
Pet shelter	132	7 (5.4)	0.8 (0.3–1.9)	0.598	6 (4.7)	1.3 (0.5–3.4)	0.617
Veterinary clinic	130	10 (7.7)	1.4 (0.6–3.2)	0.403	8 (8.0)	1.8 (0.7–4.3)	0.203
Stray	80	5 (6.3)	1.0 (0.4–2.8)	0.990	0	0 (6.7 × 10^−5^–16.9)	0.098
Clinical signs							
Diarrheic	19	1 (5.3)	0.8 (0.1–6.5)	0.860	0	0.1 (2.6×10^−4^–68.8)	0.46
Non-diarrheic	399	25 (6.3)	1.2 (0.2–9.4)		15 (3.8)	7.4 (1.4 × 10^−2^–3.8×10^3^)	
Total	418	26 (6.2)			15 (3.6)		

*Abbreviation*: n; total number of samples

* *P* < 0.05, ** *P* < 0.01

### Occurrence and risk factors of *G. duodenalis* infection in dogs and cats

*Giardia duodenalis* was detected in 60 (9.4%) of the 641 canine samples and 15 (3.6%) of the 418 feline samples (Tables 1 and 2). The infection rate in female dogs (12.4%) was significantly higher than in male dogs (6.3%; *χ*^2^ = 4.767; OR = 2.1; *P* = 0.029) (Table 1), while the infection rate in male cats (5.5%) was significantly higher than in female cats (0; *χ*^2^ = 7.349; OR = 112.2; *P* = 0.001) (Table 2). Purebred dogs (10.8%) had a higher infection rate of *G. duodenalis* than mixed breed dogs (2.4%; *χ*^2^ = 5.597; OR = 4.8; *P* = 0.018). The infection rates in household (10.1%), pet shop (10.2%) and research dogs (13.4%) were significantly higher than in dogs in veterinary clinics (2.8%; *χ*^2^ = 4.522, *P* = 0.033; *χ*^2^ = 5.051, *P* = 0.025; *χ*^2^ = 9.223, OR = 1.9, *P* = 0.017; respectively) (Table 1). Cats from veterinary clinics (8.0%) had a significantly higher infection rate than stray cats (0%; *χ*^2^ = 5.118, *P* = 0.024) (Table 2).

### Distribution of *Cryptosporidium* species

The secondary PCR products from all 44 *Cryptosporidium*-positive canine and 26 *Cryptosporidium*-positive feline samples were sequenced successfully. Among the canine samples, 42 were identified as positive for *C. canis*, and one each for *C. muris* and the *Cryptosporidium* rat genotype IV. Among the feline samples, 21 were identified as positive for *C. felis*, three for *C. parvum*, and one each for *C. muris* and *Cryptosporidium* rat genotype IV (Table 3).

**Table 3:** Species/genotypes/assemblages of *Cryptosporidium* spp. and *Giardia duodenalis* in dogs and cats by sex, breed, age, sample source and clinical signs

Variable	Dogs			Cats		
	*n*	*Cryptosporidium* genotype (*n*)	*G. duodenalis* assemblage (*n*)	*n*	*Cryptosporidium* genotype (*n*)	*G. duodenalis* assemblage (*n*)
Sex						
Female	291	*C. canis* (21); rat genotype IV (1)	C (19); D (12); C/D (4)	129	*C. felis* (10); *C. parvum* (1)	–
Male	191	*C. canis* (9)	C (4); D (6); C/D (1)	163	*C. felis* (7); *C. parvum* (1); *C. muris* (1); rat genotype IV (1)	A (1); F (8)
Unknown	159	*C. canis* (12); *C. muris* (1)	C (4); D (8)	126	*C. felis* (4); *C. parvum* (1)	F (6)
Breed						
Purebred	446	*C. canis* (28); *C. muris* (1)	C (23); D (18); C/D (5)	89	*C. felis* (8); *C. parvum* (1); *C. muris* (1); rat genotype IV (1)	F (1)
Mixed-breed	82	*C. canis* (4); rat genotype IV (1)	C (2)	187	*C. felis* (9)	F (2)
Unknown	113	*C. canis* (10)	C (2); D (8)	142	*C. felis* (4); *C. parvum* (2)	A (1); F (11)
Age (months)						
≤ 6	125	*C. canis* (14); *C. muris* (1)	C (9); D (4); C/D (1)	66	*C. felis* (7); *C. parvum* (1); rat genotype IV (1)	A (1); F (1)
> 6	402	*C. canis* (19); rat genotype IV (1)	C (16); D (14); C/D (4)	299	*C. felis* (11); *C. parvum* (1); *C. muris* (1)	F (9)
Unknown	114	*C. canis* (9)	C (2); D (8)	53	*C. felis* (3); *C. parvum* (1)	F (4)
Sample source						
Household	79	*C. canis* (11)	C (4); D (3); C/D (1)	49	*C. felis* (1)	F (1)
Pet shop	118	*C. canis* (12); *C. muris* (1)	C (8); D (3); C/D (1)	27	Rat genotype IV (1); *C. felis* (2)	
Pet shelter	134	*C. canis* (9)	C (2); D (8)	132	*C. felis* (6); *C. parvum* (1)	F (6)
Research center	201	*C. canis* (7)	C (13); D (11); C/D (3)	0		
Veterinary clinic	109	*C. canis* (3); rat genotype IV (1)	D (1)	130	*C. felis* (7); *C. parvum* (2); *C. muris* (1)	A (1); F (7)
Stray	0	–	–	80	*C. felis* (5)	–
Clinical signs						
Diarrheic	17	*C. canis* (2)	C (1); D (1)	19	*C. felis* (1)	–
Non-diarrheic	624	*C. canis* (40); *C. muris* (1); rat genotype IV (1)	C (26); D (25); C/D (5)	399	*C. felis* (20); *C. parvum* (3); *C. muris* (1); rat genotype IV (1)	A (1); F (14)
Total	641	*C. canis* (42); *C. muris* (1); rat genotype IV (1)	C (27); D (26); C/D (5)	418	*C. felis* (21); *C. parvum* (3); *C. muris* (1); rat genotype IV (1)	A (1); F (14)

Within *C. canis*, the nucleotide sequences of the *SSU* rRNA gene obtained from 21 samples were identical to the GenBank reference sequence KJ776591, while nucleotide sequences from the remaining 21 *C. canis* samples had minor differences from the reference sequence, including one single nucleotide polymorphism (SNP) in 20 samples (T to C substitution at position 627 of KJ776591), four SNPs in one sample (A to G substitution at positions 293 and 341, and T to C substitution at positions 561 and 627 of KJ776591). Within *C. felis*, the nucleotide sequences obtained from 18 samples were identical to the GenBank reference sequence KM977642, while those from the remaining three samples were identical to the reference sequence AF159113. Within *C. parvum*, two nucleotide sequences were identical to the reference sequence AB968048, whereas the third one had two SNPs compared to the reference sequence (T to C substitution at position 102, and G to A substitution at position 586 of AB968048). The nucleotide sequence from *C. muris* in the feline sample was identical to KM870575, while the one from the canine sample had two SNPs (C to G substitution at position 112, and G to A substitution at position 196). Within the *Cryptosporidium* rat genotype IV, the sequence obtained from the canine sample had two SNPs (T to C substitution at positions 360 and 418) compared to AY737582, while the other one from the feline sample had one SNP (A to G substitution at position 427) (Additional file 1: Table S1).

### Distribution of *G. duodenalis* assemblages

Fifty-eight of the 60 *G. duodenalis-*positive samples from dogs and all 15 *G. duodenalis-*positive samples from cats were sequenced successfully. There were some differences in the PCR detection rates among the *bg*, *tpi*, and *gdh* loci (Additional file 1: Table S2). *Giardia duodenalis* assemblages C, D and concurrence of both were detected in 27, 26 and 5 dogs, respectively. In contrast, assemblages A and F were found in 1 and 14 cats, respectively (Table 3). The assemblage A was identified as A1 (GenBank: L40509) at the *gdh* locus, A5 (GenBank: AB469365) at the bg locus, and A4 (GenBank: GQ329677) at the *tpi* locus. For assemblage F, the nucleotide sequences from two samples were identical to the GenBank reference sequence KX960131, and the remaining 12 samples were identical to KM977659 at the *bg* locus. The nucleotide sequences from eight samples were identical to KJ194112, and the remaining six samples were identical to AB569374 at the *gdh* locus. The nucleotide sequences from seven samples were identical to KM977655, one sample was identical to KP866788, while the remaining six samples failed in genotyping at the *tpi* locus. The nucleotide sequence differences within *G. duodenalis* assemblages C and D at the *bg*, *gdh* and *tpi* loci are shown in Additional file 1: Table S3.

### Concurrent infections of *Cryptosporidium* spp. and *G. duodenalis*

Co-infection of *Cryptosporidium* spp. and *G. duodenalis* was found in 13 dogs and 1 cat. Among them, 9 dogs had co-infection of *C. canis* and assemblage C, 4 dogs had co-infections of *C. canis* and assemblage D, and one cat had co-infection of *C. parvum* and assemblage A. The co-infection rate in household dogs (6.3%) was significantly higher than in pet shelters (0.75%; *χ*^2^ = 5.659, *P* = 0.017).

## Discussion

We have shown in the present study a common occurrence of *Cryptosporidium* spp. and *G. duodenalis* in dogs and cats in five cities in Guangdong. Young age was identified as the main risk factor for the transmission of *Cryptosporidium* spp. in these animals. The finding of higher infection rates of *Cryptosporidium* spp. in dogs and cats under 6 months is consistent with previous studies conducted elsewhere [14, 33].

Results of the present study suggest that *Cryptosporidium* spp. and *G. duodenalis* have different transmission characteristics between dogs and cats. For example, young age was identified as a risk factor for the transmission of *Cryptosporidium* spp., but not for *G. duodenalis* for both dogs and cats; pure breed was a risk factor for *Cryptosporidium* spp. in cats and *G. duodenalis* in dogs; and the female and male sexes were risk factors for *G. duodenalis* in dogs and cats, respectively. Previous studies had shown that pedigree pets were more susceptible to infectious diseases [34, 35]. Consistent with these observations, household and pet shop dogs had higher infection rate of *Cryptosporidium* spp. than dogs in research centers, while the opposite was observed for *G. duodenalis*. Some of the differences are attributable to the different life styles between dogs and cats; domestic cats mostly stay indoors and have little chance of contact with other pets or contaminated environment. In contrast, pet owners in urban areas often exercise their dogs in parks, where dogs frequently have contact with other pets and contaminated soil, increasing the risk of transmission of these parasites in household dogs [36, 37]. The higher infection rates of these parasites in pet shop and experimental dogs are expected. These places are often overcrowded with young animals and have inadequate sanitary control, which may provide a favorable environment for the fecal-oral transmission of *Cryptosporidium* spp. and *G. duodenalis* [38, 39].

Results of the present study support the suggestion that *C. canis* and *C. felis* are the most common *Cryptosporidium* species in dogs and cats, respectively [10]. As members of the five most common human-pathogenic *Cryptosporidium* species, *C. canis* and *C. felis* have been detected in humans worldwide [40–42], sometimes in both pets and their owners [43, 44]. The other zoonotic species *C. parvum* detected in three cats in the present study had been reported in pets previously [9, 45]. Subtype analysis had identified the common C. *parvum* subtypes IIaA15G2R1 and IIaA17G2R1 in urban companion animals in Great Britain [46, 47]. We have failed to subtype the *C. parvum* in the present study, thus cannot exclude the possibility of the transient passage of the parasite without established infection. The occasional infections of *C. muris* in pets are expected, as this *Cryptosporidium* species is common in rodents in China [48]. Dogs and cats are in frequent contact with rodents either in pet shops or in the wild.

*Giardia duodenalis* assemblages C, D, and F were the most prevalent genotypes in this study. These results are consistent with the observation in previous studies that these assemblages are the most common genotypes in dogs and cats [49]. In addition, assemblage A infection was detected in one cat from a veterinary clinic, and this genotype was previously found in cats in Guangzhou [18]. Even though assemblages A and B are the main zoonotic genotypes, assemblages C, D, and F have been identified in a few human cases [50–53].

## Conclusions

Results of this study suggest a common occurrence of *Cryptosporidium* spp. and *G. duodenalis* in dogs and cats in Guangdong, China, and young age, certain sex, pure breed and some living conditions could be risk factors for infections. Most *Cryptosporidium* species detected in the study, namely *C. canis*, *C. felis*, *C. parvum* and *C. muris*, are known zoonotic parasites while almost all of the *G. duodenalis* genotypes in dogs and cats are host-adapted ones. Further studies with sampling of humans and pets in the same area and characterization of zoonotic *Cryptosporidium* spp. at the subtype level are needed for improved understanding of zoonotic transmission of *Cryptosporidium* spp. in humans due to contact with pets.

## Supplementary information

**Additional file 1: Table S1.** Nucleotide substitutions in partial sequences of the *SSU* rRNA gene of *Cryptosporidium* species/genotypes obtained from dogs and cats in Guangdong. **Table S2.** Occurrence rates of *G. duodenalis* by PCR analyses of the β-giardin, glutamate dehydrogenase, and triosephosphate isomerase genes in dogs and cats. **Table S3.** Nucleotide substitutions in partial sequences of the β-giardin, glutamate dehydrogenase, and triosephosphate isomerase genes of *G. duodenalis* assemblages obtained from dogs in Guangdong.

## Data Availability

Data supporting the conclusions of this article are included within the article. Representative DNA sequences from the present study were deposited in the GenBank database under accession numbers MN272322-MN272327 for *Cryptosporidium* spp., and MN270280-MN270301 for *G. duodenalis*.

## Abbreviations

PCR: polymerase chain reaction
SSU rRNA: small subunit rRNA
*bg*: β-giardin
*gdh*: glutamate dehydrogenase
*tpi*: triosephosphate isomerase
SNP: single nucleotide polymorphism

## References

[1] Xiao L (2010). Molecular epidemiology of cryptosporidiosis: an update. Exp Parasitol..

[2] Feng Y, Xiao L (2011). Zoonotic potential and molecular epidemiology of *Giardia* species and giardiasis. Clin Microbiol Rev..

[3] Feng Y, Ryan UM, Xiao L (2018). Genetic diversity and population structure of *Cryptosporidium*. Trends Parasitol..

[4] Bouzid M, Halai K, Jeffreys D, Hunter PR (2015). The prevalence of *Giardia* infection in dogs and cats, a systematic review and meta-analysis of prevalence studies from stool samples. Vet Parasitol..

[5] Santin M (2013). Clinical and subclinical infections with *Cryptosporidium* in animals. N Z Vet J..

[6] Alves MEM, Martins FDC, Braunig P, Pivoto FL, Sangioni LA, Vogel FSF (2018). Molecular detection of *Cryptosporidium* spp. and the occurrence of intestinal parasites in fecal samples of naturally infected dogs and cats. Parasitol Res..

[7] Yang R, Ying JL, Monis P, Ryan U (2015). Molecular characterisation of *Cryptosporidium* and *Giardia* in cats (*Felis catus*) in Western Australia. Exp Parasitol..

[8] Gil H, Cano L, de Lucio A, Bailo B, de Mingo MH, Cardona GA (2017). Detection and molecular diversity of *Giardia duodenalis* and *Cryptosporidium* spp. in sheltered dogs and cats in northern Spain. Infect Genet Evol..

[9] Li W, Li Y, Song M, Lu Y, Yang J, Tao W (2015). Prevalence and genetic characteristics of *Cryptosporidium*, *Enterocytozoon bieneusi* and *Giardia duodenalis* in cats and dogs in Heilongjiang Province, China. Vet Parasitol..

[10] Lucio-Forster A, Griffiths JK, Cama VA, Xiao L, Bowman DD (2010). Minimal zoonotic risk of cryptosporidiosis from pet dogs and cats. Trends Parasitol..

[11] Xu H, Jin Y, Wu W, Li P, Wang L, Li N (2016). Genotypes of *Cryptosporidium* spp., *Enterocytozoon bieneusi* and *Giardia duodenalis* in dogs and cats in Shanghai, China. Parasit Vectors..

[12] Li J, Zhang P, Wang P, Alsarakibi M, Zhu H, Liu Y (2012). Genotype identification and prevalence of *Giardia duodenalis* in pet dogs of Guangzhou, southern China. Vet Parasitol..

[13] Li W, Liu C, Yu Y, Li J, Gong P, Song M (2013). Molecular characterization of *Giardia duodenalis* isolates from police and farm dogs in China. Exp Parasitol..

[14] Jian F, Qi M, He X, Wang R, Zhang S, Dong H (2014). Occurrence and molecular characterization of *Cryptosporidium* in dogs in Henan province, China. BMC Vet Res..

[15] Zheng G, Alsarakibi M, Liu Y, Hu W, Luo Q, Tan L (2014). Genotyping of *Giardia duodenalis* isolates from dogs in Guangdong, China based on multi-locus sequence. Korean J Parasitol..

[16] Gu YF, Wang K, Liu DY, Mei N, Chen C, Chen T (2015). Molecular detection of *Giardia lamblia* and *Cryptosporidium* species in pet dogs. Zhongguo Ji Sheng Chong Xue Yu Ji Sheng Chong Bing Za Zhi..

[17] Yang D, Zhang Q, Zhang L, Dong H, Jing Z, Li Z (2015). Prevalence and risk factors of *Giardia doudenalis* in dogs from China. Int J Environ Health Res..

[18] Zheng G, Hu W, Liu Y, Luo Q, Tan L, Li G (2015). Occurrence and molecular identification of *Giardia duodenalis* from stray cats in Guangzhou, southern China. Korean J Parasitol..

[19] Qi M, Dong H, Wang R, Li J, Zhao J, Zhang L (2016). Infection rate and genetic diversity of *Giardia duodenalis* in pet and stray dogs in Henan province, China. Parasitol Int..

[20] Zhang Y, Zhong Z, Deng L, Wang M, Li W, Gong C (2017). Detection and multilocus genotyping of *Giardia duodenalis* in dogs in Sichuan Province, China. Parasite..

[21] Yu Z, Ruan Y, Zhou M, Chen S, Zhang Y, Wang L (2018). Prevalence of intestinal parasites in companion dogs with diarrhea in Beijing, China, and genetic characteristics of *Giardia* and *Cryptosporidium* species. Parasitol Res..

[22] Anonymous. An analysis of the demographic changes in Guangdong Province in 2017. 2018. http://www.gd.gov.cn/zwgk/sjfb/sjfx/content/post_105708.html. Accessed 28 Nov 2019.

[23] Anonymous. Research and forecast report on Chinese pet market in 2014–2019. 2015. https://www.guancha.cn/society/2015_10_16_337796.shtml. Accessed 28 Nov 2019.

[24] Le XH, Wang H, Gou JZ, Chen XC, Yang GL, Yang QT (2008). Detection of *Cryptosporidium* infection among AIDS patients in Guangdong and Yunnan. Zhonghua Shi Yan He Lin Chuang Bing Du Xue Za Zhi..

[25] Ming ZF, Xi ZD, Dong CS, Serichantalergs O, Changchawalit S, Nirdnoy W (1991). Diarrhoeal disease in children less than one year of age at a children’s hospital in Guangzhou, Peopleʼs Republic of China. Trans R Soc Trop Med Hyg..

[26] Feng Y, Wang L, Duan L, Gomez-Puerta LA, Zhang L, Zhao X (2012). Extended outbreak of cryptosporidiosis in a pediatric hospital, China. Emerg Infect Dis..

[27] Wang L, Xiao L, Duan L, Ye J, Guo Y, Guo M (2013). Concurrent infections of *Giardia duodenalis*, *Enterocytozoon bieneusi*, and *Clostridium difficile* in children during a cryptosporidiosis outbreak in a pediatric hospital in China. PLoS Negl Trop Dis..

[28] Ryan U, Xiao L, Read C, Zhou L, Lal AA, Pavlasek I (2003). Identification of novel *Cryptosporidium* genotypes from the Czech Republic. Appl Environ Microbiol..

[29] Caccio SM, Beck R, Lalle M, Marinculic A, Pozio E (2008). Multilocus genotyping of *Giardia duodenalis* reveals striking differences between assemblages A and B. Int J Parasitol..

[30] Abe N, Kimata I, Iseki M (2003). Identification of genotypes of *Giardia intestinalis* isolates from dogs in Japan by direct sequencing of the PCR amplified glutamate dehydrogenase gene. J Vet Med Sci..

[31] Sulaiman IM, Fayer R, Bern C, Gilman RH, Trout JM, Schantz PM (2003). Triosephosphate isomerase gene characterization and potential zoonotic transmission of *Giardia duodenalis*. Emerg Infect Dis..

[32] Feng Y, Li N, Duan L, Xiao L (2009). *Cryptosporidium* genotype and subtype distribution in raw wastewater in Shanghai, China: evidence for possible unique *Cryptosporidium hominis* transmission. J Clin Microbiol..

[33] Scorza AV, Brewer MM, Lappin MR (2003). Polymerase chain reaction for the detection of *Cryptosporidium* spp. in cat feces. J Parasitol..

[34] Proschowsky HF, Rugbjerg H, Ersboll AK (2003). Mortality of purebred and mixed-breed dogs in Denmark. Prev Vet Med..

[35] Kim E, Choe C, Yoo JG, Oh SI, Jung Y, Cho A (2018). Major medical causes by breed and life stage for dogs presented at veterinary clinics in the Republic of Korea: a survey of electronic medical records. Peerj..

[36] Ferreira A, Alho AM, Otero D, Gomes L, Nijsse R, Overgaauw PAM (2017). Urban dog parks as sources of canine parasites: contamination rates and pet owner behaviours in Lisbon, Portugal. J Environ Public Health..

[37] Wang A, Ruch-Gallie R, Scorza V, Lin P, Lappin MR (2012). Prevalence of *Giardia* and *Cryptosporidium* species in dog park attending dogs compared to non-dog park attending dogs in one region of Colorado. Vet Parasitol..

[38] Itoh N, Itagaki T, Kawabata T, Konaka T, Muraoka N, Saeki H (2011). Prevalence of intestinal parasites and genotyping of *Giardia intestinalis* in pet shop puppies in east Japan. Vet Parasitol..

[39] Itoh N, Oohashi Y, Ichikawa-Seki M, Itagaki T, Ito Y, Saeki H (2014). Molecular detection and characterization of *Cryptosporidium* species in household dogs, pet shop puppies, and dogs kept in a school of veterinary nursing in Japan. Vet Parasitol..

[40] Feng YY, Wang L, Duan LP, Gomez-Puerta LA, Zhang LX, Zhao XK (2012). Extended outbreak of cryptosporidiosis in a pediatric hospital. China. Emerg Infect Dis..

[41] Gatei W, Wamae CN, Mbae C, Waruru A, Mulinge E, Waithera T (2006). Cryptosporidiosis: prevalence, genotype analysis, and symptoms associated with infections in children in Kenya. Am J Trop Med Hyg..

[42] Chalmers RM, Elwin K, Thomas AL, Guy EC, Mason B (2009). Long-term *Cryptosporidium* typing reveals the aetiology and species-specific epidemiology of human cryptosporidiosis in England and Wales, 2000 To 2003. Euro Surveill..

[43] Beser J, Toresson L, Eitrem R, Troell K, Winiecka-Krusnell J, Lebbad M (2015). Possible zoonotic transmission of *Cryptosporidium felis* in a household. Infect Ecol Epidemiol..

[44] Xiao LH, Cama VA, Cabrera L, Ortega Y, Pearson J, Gilman RH (2007). Possible transmission of *Cryptosporidium canis* among children and a dog in a household. J Clin Microbiol..

[45] Gharieb RMA, Merwad AMA, Saleh AA, Abd El-Ghany AM (2018). Molecular screening and genotyping of *Cryptosporidium* species in household dogs and in-contact children in Egypt: risk factor analysis and zoonotic importance. Vector Borne Zoonot Dis..

[46] Rosanowski SM, Banica M, Ellis E, Farrow E, Harwood C, Jordan B (2018). The molecular characterisation of *Cryptosporidium* species in relinquished dogs in Great Britain: a novel zoonotic risk?. Parasitol Res..

[47] Smith RP, Chalmers RM, Mueller-Doblies D, Clifton-Hadley FA, Elwin K, Watkins J (2010). Investigation of farms linked to human patients with cryptosporidiosis in England and Wales. Prev Vet Med..

[48] Feng Y, Xiao L (2017). Molecular epidemiology of cryptosporidiosis in China. Front Microbiol..

[49] Ballweber LR, Xiao L, Bowman DD, Kahn G, Cama VA (2010). Giardiasis in dogs and cats: update on epidemiology and public health significance. Trends Parasitol..

[50] Liu H, Shen Y, Yin J, Yuan Z, Jiang Y, Xu Y (2014). Prevalence and genetic characterization of *Cryptosporidium*, *Enterocytozoon*, *Giardia* and *Cyclospora* in diarrheal outpatients in China. BMC Infect Dis..

[51] Strkolcova G, Mad’ar M, Hinney B, Goldova M, Mojzisova J, Halanova M (2015). Dogʼs genotype of *Giardia duodenalis* in human: first evidence in Europe. Acta Parasitol..

[52] Broglia A, Weitzel T, Harms G, Caccio SM, Nockler K (2013). Molecular typing of *Giardia duodenalis* isolates from German travellers. Parasitol Res..

[53] Gelanew T, Lalle M, Hailu A, Pozio E, Caccio SM (2007). Molecular characterization of human isolates of *Giardia duodenalis* from Ethiopia. Acta Trop..

