# Supplementary material for: Genetic characterization of Cryptosporidium spp. and Giardia duodenalis in dogs and cats in Guangdong, China
Source: Parasit Vectors. 2019 Nov 29;12:571. doi: 10.1186/s13071-019-3822-z (PMC6884805; doi:10.1186/s13071-019-3822-z)
Supplement: Supplementary file 1 — Additional file 1: Table S1. Nucleotide substitutions in partial sequences of the SSU rRNA gene of Cryptosporidium species/genotypes obtained from dogs and cats in Guangdong. Table S2. Occurrence rates of G. duodenalis by PCR analyses of the β-giardin, glutamate dehydrogenase, and triosephosphate isomerase genes in dogs and cats. Table S3. Nucleotide substitutions in partial sequences of the β-giardin, glutamate dehydrogenase, and triosephosphate isomerase genes of G. duodenalis assemblages obtained from dogs in Guangdong. [file 13071_2019_3822_MOESM1_ESM.docx]

**Additional file 1: Table S1.** Nucleotide substitutions in partial **s**equences of the small subunit rRNA gene of *Cryptosporidium* species/genotypes obtained from dogs and cats in Guangdong.

| **Host** | **Species/genotypes** | **GenBank**  **accession Nos. (n )^a^** | **Nucleotide at position ^b^** | | | | | | | | | | |
| --- | --- | --- | --- | --- | --- | --- | --- | --- | --- | --- | --- | --- | --- |
|  |  |  | 102 | 112 | 196 | 293 | 360 | 341 | 418 | 427 | 561 | 586 | 627 |
| Dogs | *C. canis* | Ref**.** (KJ776591) |  |  |  | A |  | A |  |  | T |  | T |
|  |  | MN272326 (20) |  |  |  | **.** |  | **.** |  |  | **.** |  | C |
|  |  | MN272327 (1) |  |  |  | G |  | G |  |  | C |  | C |
|  | *C. muris* | Ref. (KM870575) |  | C | G |  |  |  |  |  |  |  |  |
|  |  | MN272323 (1) |  | G | A |  |  |  |  |  |  |  |  |
|  | *Cryptosporidium* rat genotype IV | Ref. (AY737582) |  |  |  |  | T |  | T |  |  |  |  |
|  |  | MN272324 (1) |  |  |  |  | C |  | C |  |  |  |  |
| Cats | *C. parvum* | Ref. (AB968048) | T |  |  |  |  |  |  |  |  | G |  |
|  |  | MN272322 (1) | C |  |  |  |  |  |  |  |  | A |  |
|  | *Cryptosporidium* rat genotype IV | Ref. (AY737582) |  |  |  |  |  |  |  | A |  |  |  |
|  |  | MN272325 (1) |  |  |  |  |  |  |  | G |  |  |  |

The nucleotide identical to the one in the reference sequence is represented by a dot.

^a^ Numbers in the parentheses are number of specimens with the sequence type.

^b^ Nucleotide position numbers of the reference (Ref.) sequences.

**Additional file 1: Table S2.** Occurrence rates of *Giardia duodenalis* by PCR analyses of the β-giardin, glutamate dehydrogenase, and triosephosphate isomerase gene in dogs and cats.

| **Genetic loci** | **Dogs** | | |  | **Cats** | | |
| --- | --- | --- | --- | --- | --- | --- | --- |
|  | **No.** | **Positive no. (%)** | ***G. duodenalis* assemblage (no.)** |  | **No.** | **Positive no. (%)** | ***G. duodenalis* assemblage (no.)** |
| *bg* | 641 | 60 (9.4) | C (27), D (31), mixed (2) |  | 418 | 15 (3.6) | F (14), A (1) |
| *tpi* | 641 | 49 (7.6) | C (22), D (27) |  | 418 | 9 (2.2) | F (8), A (1) |
| *gdh* | 641 | 46 (7.2) | C (25), D (21) |  | 418 | 15 (3.6) | F (14), A (1) |

**Additional file 1: Table S3.** Nucleotide substitutions in partial sequences of the β-giardin, glutamate dehydrogenase, and triosephosphate isomerase genes of *Giardia duodenalis* assemblages obtained from dogs in Guangdong.

| **Genetic loci** | **Assemblages** | **GenBank**  **accession Nos. (n) ^a^** | **Nucleotide at position ^b^** | | | | | | | | | | | | | | | | |
| --- | --- | --- | --- | --- | --- | --- | --- | --- | --- | --- | --- | --- | --- | --- | --- | --- | --- | --- | --- |
|  |  |  | 33 | 38 | 39 | 81 | 99 | 100 | 135 | 146 | 271 | 316 | 318 | 348 | 368 | 369 | 379 | 393 | 424 |
| *tpi* | Assemblage C | Ref**.** (MF974556) | T |  |  |  |  |  | G |  |  |  |  |  |  |  |  |  |  |
|  |  | MN270280 (1) | C |  |  |  |  |  | A |  |  |  |  |  |  |  |  |  |  |
|  | Assemblage C | Ref**.** (KP866787) |  |  |  |  |  | T |  |  |  |  |  | G |  |  |  |  | A |
|  |  | MN270281 (1) |  |  |  |  |  | C |  |  |  |  |  | A |  |  |  |  | G |
|  | Assemblage C | Ref**.** (KY448466) |  |  |  |  |  | T |  | G | G | T |  | G |  | T | A |  |  |
|  |  | MN270285 (1) |  |  |  |  |  | **.** |  | A | A | **.** |  | A |  | **.** | **.** |  |  |
|  |  | MN270283 (1) |  |  |  |  |  | C |  | **.** | **.** | C |  | **.** |  | **.** | **.** |  |  |
|  |  | MN270282 (1) |  |  |  |  |  | C |  | **.** | **.** | C |  | **.** |  | C | **.** |  |  |
|  |  | MN270284 (1) |  |  |  |  |  | **.** |  | **.** | **.** | **.** |  |  |  | **.** | C |  |  |
|  | Assemblage C | Ref. (KT728510) |  |  |  |  | C |  |  |  |  |  |  |  | C |  |  | A |  |
|  |  | MN270286 (2) |  |  |  |  | T |  |  |  |  |  |  |  | T |  |  | G |  |
|  |  | MN270287 (2) |  |  |  |  | **.** |  |  |  |  |  |  |  | T |  |  | G |  |
|  | Assemblage D | Ref. (JN587488) |  | T | T | C |  |  |  |  |  |  | T |  |  |  | C |  |  |
|  |  | MN270291 (1) |  | C | **.** | T |  |  |  |  |  |  | **.** |  |  |  | **.** |  |  |
|  |  | MN270290 (1) |  | **.** | C | **.** |  |  |  |  |  |  | **.** |  |  |  | T |  |  |
|  |  | MN270292 (1) |  | **.** | **.** | **.** |  |  |  |  |  |  | C |  |  |  | T |  |  |
|  |  | MN270288 (5) |  | **.** | **.** | **.** |  |  |  |  |  |  | C |  |  |  | **.** |  |  |
|  |  | MN270289 (1) |  | **.** | **.** | **.** |  |  |  |  |  |  | **.** |  |  |  | T |  |  |
|  |  |  |  |  |  |  |  |  |  |  |  |  |  |  |  |  |  |  |  |
|  |  |  | 48 | 87 | 127 | 130 | 14112 | 205 | 207 | 217 | 231 | 232 | 319 |  |  |  |  |  |  |
| *bg* | Assemblage C | Ref. (KY979498) |  |  |  |  |  |  |  |  | G |  |  |  |  |  |  |  |  |
|  |  | MN270293 (2) |  |  |  |  |  |  |  |  | A |  |  |  |  |  |  |  |  |
|  | Assemblage C | Ref. (KY979499) |  |  |  |  |  | C |  |  |  |  |  |  |  |  |  |  |  |
|  |  | MN270294 (1) |  |  |  |  |  | T |  |  |  |  |  |  |  |  |  |  |  |
|  | Assemblage C | Ref. (JN416513) |  |  |  |  |  |  | T |  |  |  |  |  |  |  |  |  |  |
|  |  | MN270295 (1) |  |  |  |  |  |  | C |  |  |  |  |  |  |  |  |  |  |
|  | Assemblage C | Ref. (JN416552) |  |  |  |  |  |  |  | T |  | G |  |  |  |  |  |  |  |
|  |  | MN270296 (1) |  |  |  |  |  |  |  | C |  | **.** |  |  |  |  |  |  |  |
|  |  | MN270297 (2) |  |  |  |  |  |  |  | C |  | A |  |  |  |  |  |  |  |
|  | Assemblage D | Ref. (KY979497) |  |  |  |  |  |  |  |  |  |  | G |  |  |  |  |  |  |
|  |  | MN270298 (1) |  |  |  |  |  |  |  |  |  |  | A |  |  |  |  |  |  |
|  |  |  |  |  |  |  |  |  |  |  |  |  |  |  |  |  |  |  |  |
|  |  |  | 48 | 87 | 127 | 130 | 141A | 249 | 261 | 372 | 468 |  |  |  |  |  |  |  |  |
| *gdh* | Assemblage C | Ref. (MF990015) |  |  |  |  | C |  |  |  |  |  |  |  |  |  |  |  |  |
|  |  | MN270299 (1) |  |  |  |  | T |  |  |  |  |  |  |  |  |  |  |  |  |
|  | Assemblage D | Ref. (EF507619) |  |  |  |  |  |  | G | G |  |  |  |  |  |  |  |  |  |
|  |  | MN270300 (1) |  |  |  |  |  |  | A | A |  |  |  |  |  |  |  |  |  |
|  | Assemblage D | Ref. (EF507634) |  |  |  |  |  | C |  |  | A |  |  |  |  |  |  |  |  |
|  |  | MN270301 (1) |  |  |  |  |  | T |  |  | T |  |  |  |  |  |  |  |  |

The nucleotide identical to the one in the reference sequence is represented by a dot.

^a^ Numbers in the parentheses are number of specimens with the sequence type.

^b^ Nucleotide position numbers of the reference (Ref.) sequences.
